# Anaerobic digestion performance of sweet potato vine and animal manure under wet, semi-dry, and dry conditions

**DOI:** 10.1186/s13568-018-0572-9

**Published:** 2018-03-22

**Authors:** Enlan Zhang, Jiajia Li, Keqiang Zhang, Feng Wang, Houhua Yang, Suli Zhi, Guangqing Liu

**Affiliations:** 1 Biomass Energy and Environmental Engineering Research Center, College of Chemical Engineering, Beijing University of Chemical Technology, Beijing, 100029 China; 2 Innovation Team of Animal Husbandry Pollution Prevention and Control, Agro-Environmental Protection Institute, Chinese Academy of Agricultural Sciences, 504 Zonghe Building, No.31 Fukang Road, Nankai District, Tianjin, 300191 People’s Republic of China

**Keywords:** Sweet potato vine, Animal manure, Total solid, Co-digestion, Process stability

## Abstract

Sweet potato vine (SPV) is an abundant agricultural waste, which is easy to obtain at low cost and has the potential to produce clean energy via anaerobic digestion (AD). The main objectives of this study were to reveal methane production and process stability of SPV and the mixtures with animal manure under various total solid conditions, to verify synergetic effect in co-digestion of SPV and manure in AD systems, and to determine the kinetics characteristics during the full AD process. The results showed that SPV was desirable feedstock for AD with 200.22 mL/g VS_added_ of methane yield in wet anaerobic digestion and 12.20 L_methane_/L_working volume_ in dry anaerobic digestion (D-AD). Synergistic effects were found in semi-dry anaerobic digestion and D-AD with each two mixing feedstock. In contrast with SPV mono-digestion, co-digestion with manure increased methane yield within the range of 14.34–49.11% in different AD digesters. The values of final volatile fatty acids to total alkalinity (TA) were below 0.4 and the values of final pH were within the range of 7.4–8.2 in all the reactors, which supported a positive relationship between carbohydrate hydrolysis and methanogenesis during AD process. The mathematical modified first order model was applied to estimate substrate biodegradability and methane production potential well with conversion constant ranged from 0.0003 to 0.0953 1/day, which indicated that co-digestion increased hydrolysis efficiency and metabolic activity. This work provides useful information to improve the utilization and stability of digestion using SPV and livestock or poultry manure as substrates.

## Introduction

Total solid (TS) concentration is one of the most important parameters in the efficiency assessment of anaerobic digestion. It is widely accepted including wet, semi-dry, and dry anaerobic digestion, when TS of substrate are < 10, 10–15, or > 15%, respectively (Li et al. 2011; Liotta et al. 2015). Wet anaerobic digestion (W-AD) is widely applied to treat livestock and poultry breeding wastewater, food waste and energy crop due to high methane yield per unit substrate, low level of sludge generation and convenient operation and maintenance (Zhang et al. 2000; Demirel and Scherer 2009; Nagao et al. 2012). However, for feedstock with low moisture content, such as crop straw and municipal sludge, dry anaerobic digestion (D-AD) is a better choice because of low consumption of water, small reactor requirement and high volumetric methane production (Guendouz et al. 2010; Brown and Li 2013). In addition, the storage and recovery of anaerobic sludge activity has aroused the concern of researchers (Li et al. 2014), which help to solve the demand for large amounts of activated sludge and accelerate the start-up of D-AD reactors.

Traditionally, animal manure was used as mono-substrate to produce renewable biogas in most of the digesters around the world (Wu et al. 2010). However, the deficiency of carbon may hinder the biodegradability of substrate and decrease the methane yield. Compared with mono-substrate digestion, co-digestion has many benefits including dilution of potential toxic compounds, synergistic effects of microorganisms, improved balance of nutrients, increased digestion rate and better biogas yield (Sosnowski et al. 2003; Cuetos et al. 2011). Many studies have been reported on anaerobic co-digestion with crop stalk and animal manure (Søndergaard et al. 2015; Awais et al. 2016; Hassan et al. 2017). The substrate selection for co-digestion depends from the amount of feedstock and the cost of collection and transportation (Asam et al. 2011). Sweet potato vine (SPV) and animal manure are typical agricultural wastes in the world, especially in China. The annual production of animal manure has exceeded 2.1 billion tons since 2011 in China (Zhu and Ma 2014). Meanwhile, 106.64 million tons of sweet potato was harvested in 2015 (National Bureau of Statistics of China 2016), which was 53.32 million tons of fresh SPV calculated by the shoot–root ratio of 0.5. So far, there is no literature on the evaluation of methane productivity and operation stability of SPV alone and co-digestion from SPV and animal manure in W-AD, semi-dry anaerobic digestion (SD-AD) or D-AD systems.

The objectives of the present study were to: (1) investigate the methane yield, volumetric methane productivity and process stability during the digestion of SPV and the mixtures with animal manure; (2) verify the synergetic effect in the co-digestion system under various TS conditions; (3) determine the dynamical features using modified first order model during the full AD process.

## Methods

### Substrates and inoculum

Fresh dairy manure (DM), chicken manure (CM) and pig manure (PM) were obtained from large-scale farms in Binhai New District and Ninghe District, Tianjin, China. Sweet potato vine (SPV) was collected in Changping District, Beijing, China. SPV was dried in air for 1 week and then smashed to 20 meshes by a mill (Taisite, China). Inoculum used in this study was activated sludge from a running anaerobic digester treating pig manure, which locates in Xiqing District, Tianjin, China. Before utilization, inoculum was passed a 2 mm sieve to separate and discard large particles and then partially pre-concentrated by a centrifuge (Xiangyi, China). The characteristics of substrates and inoculum are presented in Table 1.

**Table 1:** Characteristics of substrates and inoculum

Parameter	SPV	DM	PM	CM	OS	CS
TS (%)^a^	91.8 ± 0.7	20.2 ± 0.1	31.6 ± 0.2	28.2 ± 0.3	3.8 ± 0.0	20.7 ± 0.6
VS (%)^a^	78.7 ± 0.9	17.0 ± 0.4	24.2 ± 0.3	17.3 ± 0.3	2.0 ± 0.1	11.6 ± 0.5
VS/TS (%)	85.7 ± 0.4	83.9 ± 1.6	76.5 ± 0.8	61.4 ± 0.7	52.6 ± 1.3	56.1 ± 0.5
C (%)^b^	41.4 ± 1.0	43.5 ± 0.2	40.0 ± 0.1	31.4 ± 0.8	30.1 ± 0.9	30.1 ± 0.9
H (%)^b^	5.0 ± 0.2	6.0 ± 0.4	5.5 ± 0.5	4.3 ± 0.3	4.3 ± 0.2	4.3 ± 0.2
N (%)^b^	2.7 ± 0.0	2.7 ± 0.2	3.6 ± 0.1	4.1 ± 0.1	4.0 ± 0.1	4.0 ± 0.1
C/N	15.1 ± 0.6	16.1 ± 1.2	11.2 ± 0.4	7.7 ± 0.1	7.5 ± 0.0	7.5 ± 0.0
pH	ND	8.2 ± 0.1	6.5 ± 0.0	7.2 ± 0.0	8.1 ± 0.0	8.8 ± 0.0
TA (mg CaCO_3_/g)^b^	ND	33.4 ± 0.2	65.5 ± 3.4	53.0 ± 1.6	5.7 ± 0.0	24.7 ± 0.4
VFAs (g/kg)^b^	0.1 ± 0.0	0.1 ± 0.0	2.0 ± 0.2	0.5 ± 0.1	0.2 ± 0.0	0.2 ± 0.0
TAN (mg/g)^b^	ND	2.4 ± 0.0	12.1 ± 0.5	5.5 ± 0.1	ND	ND

ND, not determined; SPV, sweet potato vine; DM, dairy manure; PM, pig manure; CM, chicken manure; OS, original sludge; CS, centrifuged sludge; TS: total solid; VS: volatile solid; TA, total alkalinity; VFAs, volatile fatty acid; TAN, total ammonia–nitrogen

^a^As total weight of sample

^b^As TS of sample

### Test setup

Batch AD tests were carried out in triplicate using 1 L Duran glass bottles with a working volume of 0.5 L at 37 °C in an incubate room. TS is made up of volatile solid (VS) and ash, and only VS can be degraded and converted into methane. To improve the evaluation accuracy of volumetric methane productivities and unit substrate utilization efficiency, calculation based on VS was used in this study. The initial organic loading (OL) for W-AD, SD-AD and D-AD was 30, 60 and 90 g-VS/L, respectively. The substrate to inoculum (S/I) ratio of each digester was 3 on VS basis. Substrates were mixed with tap water and inoculum, and the final TS were 5.49–6.93% for W-AD; 10.49–13.36% for SD-AD; and 15.73–20.04% for D-AD (Table 2). All treatments were conducted at the same time including mono-digestion of SPV or animal manure and co-digestion of SPV and manure at 1:1 ratio on VS basis. The digesters were tightly closed with rubber stopper and screw caps, and then argon gas was used to purge the headspace of digesters to ensure an initial anaerobic environment. Inoculum and tap water without any substrate addition was used as blank to correct the methane yield. Each digester was shaken manually twice a day for 30 s. The total digestion time of each digester was 40 days.

**Table 2:** The initial operating parameters of the anaerobic reactors

Samples	W-AD		SD-AD		D-AD	
	OL (g-VS/L)	TS (%)	OL (g-VS/L)	TS (%)	OL (g-VS/L)	TS (%)
SPV	30	5.49	60	10.49	90	15.73
DM	30	5.55	60	10.59	90	15.89
SPV+DM	30	5.52	60	10.54	90	15.81
PM	30	5.94	60	11.38	90	17.07
SPV+PM	30	5.72	60	10.93	90	16.40
CM	30	6.93	60	13.36	90	20.04
SPV+CM	30	6.21	60	11.92	90	17.88

OL, organic loading; VS, volatile solid; TS, total solid

### Analytical methods

TS and VS were measured by using standard methods described in APHA (2005). Elemental compositions of substrates were determined with an elemental analyzer (Vario EL cube, Germany). pH value was measured with a Delta 320 pH electrode (Mettler Toledo, USA). Total alkalinity (TA) value was determined using ET 18 alkalimeter (Mettler Toledo, USA) with 0.1 mol/L of HCl solution as neutralizer. Total ammonia–nitrogen (TAN) concentration was analyzed using Kjeldahl determination with semi-automatic Kjeldahl apparatus (Ketuo, China). Free ammonia (FA) concentration in the liquid was calculated from TAN concentration according to the following formula (1) (Rajagopal et al. 2013):

1 $$FA = TAN\, \times \,\left( {1\, + \,\frac{{10^{ - pH} }}{{10^{{ - \,\left( {0.09018\, + \,\frac{2729.92}{T(K)}} \right)}} }}} \right)^{ - 1}$$

where T represents the absolute temperature and pH is the final pH of sample.

The pressure in the bottle headspace was measured by a pressure gauge (WAL Mess-und Regelsysteme GmbH, Germany) at 37 °C under atmospheric pressure by inserting the syringe needle through the robber stopper. After the biogas in the headspace was released, the pressure was measured again. The following formula (2) was used to calculate the biogas yield (ElMashad and Zhang 2010):

2 $$V_{biogas} \, = \,\Delta P\, \times \,V_{head} \, \times \,{C \mathord{\left/ {\vphantom {C {(R\, \times \,T)}}} \right. \kern-0pt} {(R\, \times \,T)}}$$

where *V*_*biogas*_ refers to daily biogas volume (mL), Δ*P* means absolute pressure different (mbar), *V*_*head*_ represents volume of the head space (mL), *C* stands for molar volume (22.41 L/mol), *T* is absolute temperature (K), and R represents universal gas constant (83.14 L/mbar/K/mol).

Biogas composition was measured using gas chromatography (Thermo Fisher, USA) with automatic sampler as described by Li et al. (2014). Volatile fatty acids (VFAs) in digestate were determined by gas chromatography (Thermo Fisher, USA) with automatic sampler as described by Song et al. (2016).

### Kinetic modeling

The mathematical modified first order model (3) (Vavilin et al. 2008), which includes parameters for ultimate methane yield and conversion constant, has been widely applied in simulating the entire AD process (Zhao et al. 2016).

3 $$Y\, = \,Y_{0} \left[ {\left( {1 - \beta } \right)\, - \,(1 - \beta )\,{ \exp }( - \kappa t)} \right]$$

where Y refers to the cumulative methane yield (mL/g VS_added_), Y_0_ is the ultimate methane yield (mL/g VS_added_), *β* represents the non-degradable fraction of the substrate, *κ* stands for the rate constant (1/day), and t refers to the digestion time (day).

### Statistical analysis

All the experimental data in this study was analyzed statistically using one way analysis of variance (ANOVA) with IBM SPSS Statistics 22 (IBM, USA). To conduct pairwise comparisons of the average of each studied parameter, the Fisher Least Significant Difference (LSD) was calculated at α = 0.01 and α = 0.05 (Zhang et al. 2007).

## Results

### Characterization of substrates and inoculum

The characterization of substrates and inoculum are shown in Table 1. SPV contained higher TS and VS content than manure. The VS/TS ratio of SPV, DM, PM, and CM were 85.7, 83.9, 76.5 and 61.4%, respectively. The ratio of C/N of CM was determined to be 7.7, which was the lowest one in four types of feedstock. PM contained the highest TA and TAN concentration among animal manure.

### Daily methane production performance

**Fig. 1:**
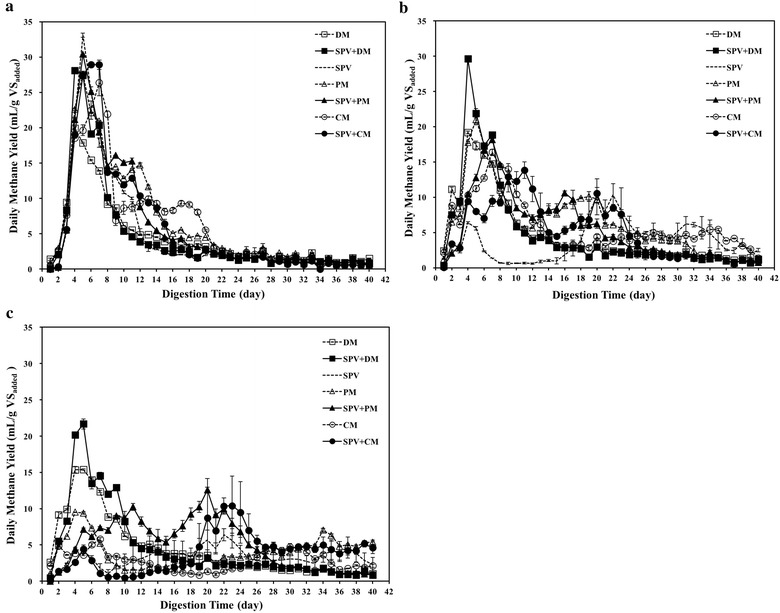
Daily methane production performance from SPV and the mixtures with animal manure in **a** W-AD, **b** SD-AD and **c** D-AD systems

The daily methane yield of SPV, animal manure and their mixtures under W-AD, SD-AD and D-AD are shown in Fig. 1. The methane production started immediately, and similar trends were observed for combinations of SPV and manure in W-AD systems. Anaerobic digestion of SPV alone showed significantly (*p < *0.05) increase of daily methane yield in W-AD than in SD-AD and D-AD in the first 2 weeks. The highest daily methane yields of mono-digestion of SPV were 33.08, 10.33 and 6.39 mL/g VS_added_ in W-AD, SD-AD and D-AD, respectively. In mono-digestion treatments of animal manure, all the peaks of daily methane yield were obtained from day 4 to day 7. The highest daily methane yield of DM, PM and CM alone were 19.91, 27.57 and 26.37 mL/g VS_added_ in W-AD; 19.19, 21.00 and 16.39 mL/g VS_added_ in SD-AD; 15.38, 9.50 and 5.79 mL/g VS_added_ in D-AD, respectively. Compared four types of substrates, the changes of TS concentration had the least effect to daily methane production performance of DM.

For co-digestion of SPV with DM, PM and CM, the highest daily methane yields were 28.08, 30.38 and 28.95 mL/g VS_added_ in W-AD; 29.62, 18.14 and 13.86 mL/g VS_added_ in SD-AD; 21.67, 12.55 and 10.39 mL/g VS_added_ in D-AD, respectively. The results showed that co-digestion increased the daily methane yield of manure under the same state. As TS concentration was over 10%, similar trends were found that the decrease of the daily methane yield in SPV+DM digesters was far less than in SPV+PM and SPV+CM systems.

### Comparison of methane yield and productivity

**Fig. 2:**
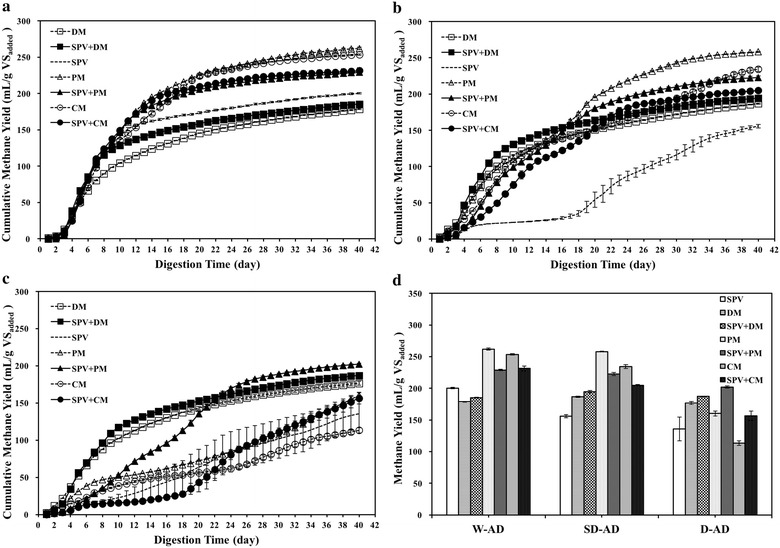
Comparison of cumulative methane yield from SPV and the mixtures with animal manure in **a** W-AD, **b** SD-AD, **c** D-AD and **d** comparison of methane yield systems

Figure 2 shows the cumulative methane yield of SPV, animal manure and their mixtures under different TS conditions. Almost 90% of the experimental methane yields were obtained after 28, 35 and 37 days in W-AD, SD-AD and D-AD, respectively (Fig. 2a–c). In the first 2 weeks, fermentation suppressions were obviously found for SPV in SD-AD and D-AD. The cumulative methane yields of mono-digestion of SPV were 155.69 and 135.51 mL/g VS_added_ in SD-AD and D-AD, which were 77.77 and 67.68% of the yield in W-AD, respectively. In view of feedstock characterization, mono-digestion of SPV obtained significantly (*p < *0.05) higher methane yield than DM in W-AD, but lower yield (*p* *<* 0.01) than animal manure in SD-AD. In W-AD and SD-AD, mono-digestion with PM represented the highest methane yield, which was 261.88 and 258.02 mL/g VS_added_, respectively. In D-AD, co-digestion from SPV and PM obtained the methane peak at 202.06 mL/g VS_added_.

As revealed in Fig. 2d, the results indicated that total solid had negative effect on substrate utilization, especially in mono-digestion reactors. Compared to W-AD treatments, the cumulative methane yields of SD-AD and D-AD were reduced by 1.17–55.34%. However, co-digestion improved the methane production of substrate per unit, especially for SPV+DM. In comparison with SPV under the same TS condition, manure addition had an increase of methane yield with 14.34–49.11% except SPV+DM in W-AD.

**Fig. 3:**
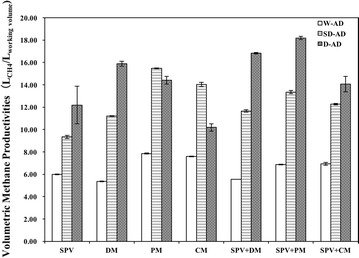
Comparison of volumetric methane productivities from different mixtures of SPV and animal manure under W-AD, SD-AD and D-AD conditions

Figure 3 shows the comparison of volumetric methane productivities (VMP) in W-AD, SD-AD and D-AD reactors. All of digesters in D-AD showed higher VMP than in W-AD and SD-AD, except PM and CM mono-digestion. The volumetric methane yields were 5.36–7.86 L_methane_/L_working_ _volume_ in W-AD; 9.34–15.48 L_methane_/L_working_ _volume_ in SD-AD; 10.19–18.19 L_methane_/L_working_ _volume_ in D-AD, respectively. D-AD improved volumetric methane productivities by 55.52–109.83% of W-AD, whereas SD-AD increased VMP by 33.98–202.83% of W-AD.

### Synergistic effect of co-digestion

Synergistic effect could be seen as an additional methane yield for co-digestion over the weighted average of the individual methane yield of each feedstock (Esposito et al. 2012). Weighted experimental methane yield (weighted EMY) was calculated as follows (4):

4 $${\text{Weighted}}\;{\text{EMY}}\, = \,{\text{EMY}}_{\text{SPV}} \, \times \,0. 5\, + \,{\text{EMY}}_{\text{manure}} \, \times \,0. 5$$

where Weighted EMY refers to the weighted average of experimental methane yield for co-substrates, EMY_SPV_ and EMY_manure_ stand for the experimental methane yield for SPV and animal manure, respectively.

**Fig. 4:**
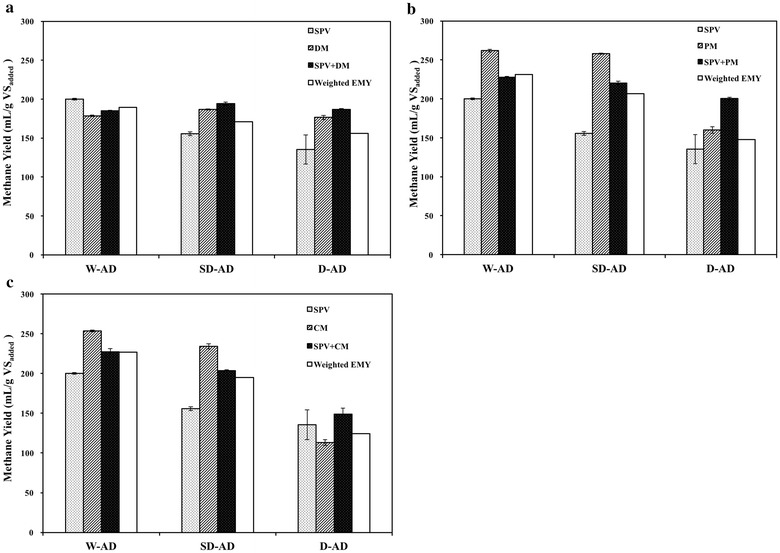
Evaluation of synergistic effect of co-digestion with SPV and animal manure under various total solid conditions. **a** SPV+DM, **b** SPV+PM, **c** SPV+CM

If the difference (EMY-the standard deviation absolute value) was bigger than Weighted EMY, synergistic effect was confirmed to be available. The evaluation of synergistic effect on different condition of digestions was represented in Fig. 4. Synergistic effect was confirmed in co-digestion of SPV and three types of manure in SD-AD and D-AD. Manure addition improved methane yield by 13.53 and 19.85% of weighted TMY for DM, 7.62 and 36.69% for PM, 5.09 and 25.71% for CM, respectively. Therefore, co-digestion of SPV and manure under SD-AD or D-AD is a recommendable way to produce biogas with small reactor volume.

### Evaluation of process stability in wet, semi-dry and dry digestion

Imbalances of hydrolytic bacteria, acetogenic bacteria and methanogenic archaea may decrease methane productivity, even fail the fermentation. Usually, VFAs accumulation and ammonia inhibition are the significant factors to bring process stability down. The parameters of process stability for all the treatments are shown in Table 3.

**Table 3:** Characterization of digestate from different mixtures of SPV and animal manure in various anaerobic conditions

Samples	Final pH	Final TAN (mg/g)	Final FA (mg/kg)	Final VFAs (g/kg)	Final TA (mg CaCO_3_/g)	VFAs/TA
W-AD						
CK	7.8 ± 0.0	1.1 ± 0.1	37.2 ± 1.9	0.1 ± 0.0	3.4 ± 0.4	0.0 ± 0.0
SPV	7.9 ± 0.0	1.7 ± 0.1	67.6 ± 2.7	0.5 ± 0.0	8.7 ± 0.4	0.1 ± 0.0
DM	7.8 ± 0.0	1.3 ± 0.2	41.4 ± 7.5	1.2 ± 0.0	7.4 ± 0.1	0.2 ± 0.0
SPV+DM	7.8 ± 0.0	1.4 ± 0.1	52.2 ± 4.2	0.1 ± 0.0	7.9 ± 0.4	0.0 ± 0.0
PM	7.6 ± 0.0	1.8 ± 0.7	44.2 ± 16.1	0.3 ± 0.0	10.7 ± 0.1	0.0 ± 0.0
SPV+PM	7.6 ± 0.0	1.8 ± 0.0	39.3 ± 0.2	0.3 ± 0.0	9.0 ± 0.4	0.0 ± 0.0
CM	7.8 ± 0.0	3.0 ± 0.0	113.7 ± 0.8	0.5 ± 0.1	13.0 ± 0.1	0.0 ± 0.0
SPV+CM	7.7 ± 0.0	2.3 ± 0.1	68.7 ± 2.7	1.4 ± 0.1	11.1 ± 0.1	0.1 ± 0.0
SD-AD						
CK	7.4 ± 0.0	0.7 ± 0.2	9.3 ± 2.2	0.1 ± 0.0	4.0 ± 0.1	0.0 ± 0.0
SPV	7.7 ± 0.0	1.6 ± 0.0	42.0 ± 0.7	0.8 ± 0.1	10.6 ± 0.1	0.1 ± 0.0
DM	7.3 ± 0.0	1.3 ± 0.2	14.7 ± 1.9	0.8 ± 0.1	10.0 ± 0.1	0.1 ± 0.0
SPV+DM	7.5 ± 0.0	1.6 ± 0.1	26.0 ± 0.9	2.6 ± 0.2	11.2 ± 0.1	0.2 ± 0.0
PM	7.6 ± 0.1	2.9 ± 0.0	57.7 ± 0.7	0.7 ± 0.1	16.6 ± 0.1	0.0 ± 0.0
SPV+PM	7.8 ± 0.0	2.3 ± 0.5	73.3 ± 17.4	0.4 ± 0.0	15.1 ± 0.1	0.0 ± 0.0
CM	8.1 ± 0.0	4.5 ± 0.0	306.1 ± 0.6	0.2 ± 0.0	22.7 ± 0.1	0.0 ± 0.0
SPV+CM	8.0 ± 0.0	3.4 ± 0.0	171.3 ± 2.1	1.3 ± 0.1	16.9 ± 0.1	0.1 ± 0.0
D-AD						
CK	7.7 ± 0.1	1.4 ± 0.1	38.3 ± 2.1	0.2 ± 0.0	6.7 ± 0.1	0.0 ± 0.0
SPV	7.9 ± 0.0	3.2 ± 0.1	139.7 ± 2.7	4.3 ± 0.2	17.9 ± 0.3	0.3 ± 0.0
DM	7.4 ± 0.0	2.5 ± 0.0	37.3 ± 0.2	3.8 ± 0.1	15.9 ± 0.0	0.2 ± 0.0
SPV+DM	7.8 ± 0.1	2.4 ± 0.6	77.3 ± 20.3	2.3 ± 0.1	17.1 ± 0.3	0.1 ± 0.0
PM	8.2 ± 0.1	4.9 ± 0.1	420.8 ± 11.0	0.8 ± 0.1	26.7 ± 0.1	0.0 ± 0.0
SPV+PM	7.9 ± 0.1	4.0 ± 0.4	187.9 ± 18.3	0.5 ± 0.0	23.5 ± 1.0	0.0 ± 0.0
CM	8.1 ± 0.1	6.7 ± 0.5	444.1 ± 32.9	0.5 ± 0.0	30.3 ± 0.2	0.0 ± 0.0
SPV+CM	8.2 ± 0.1	4.9 ± 0.1	382.3 ± 11.0	1.8 ± 0.2	24.6 ± 0.3	0.1 ± 0.0

CK, control treatment; SPV, sweet potato vine; DM, dairy manure; PM, pig manure; CM, chicken manure; W-AD, wet anaerobic digestion; SD-AD, semi-dry anaerobic digestion; D-AD, dry anaerobic digestion; TA, total alkalinity; TAN, total ammonia nitrogen; FA, free ammonia; VFAs, volatile fatty acids

The value of pH is often considered as an important and accessible criterion to evaluate AD stability. The reactors in this study showed the final pH value within the range of 7.4–8.2, which indicated an acceptable performance under various TS conditions, especially for D-AD (Lay et al. 1997; Weiland 2010). Usually, the ratio of VFAs to TA is a complementary parameter to judge whether methanogenic archaea was restrained in AD reactors. As seen in Table 3, generally, with the total solid increasing, the TA value highlighted rising trends. In the same type of reactors with similar TS concentration, the TA value of chicken manure system and pig manure system were higher than dairy manure system, which was in keeping with the characteristics of materials as shown in Table 1. Although suppression phenomenon of methane production was found in the beginning of the digestion, especially in D-AD, there was no obvious VFAs accumulation found in the end of AD tests. The final VFAs concentration was in the range of 0.2–4.3 g/kg in D-AD, which was higher and slight higher than in W-AD and SD-AD. The further consumption of VFAs during the later period of digestion broke down the inhibition aggravation of gas production, improved daily methane production (Fig. 1b, c), and resulted in a satisfactory volumetric methane production (Fig. 3). Meanwhile, the values of VFAs to TA were very low because of low VFAs residual in the end of digestion. All the data of VFAs to TA was below 0.4 in different AD reactors, which supported a positive relationship between carbohydrate hydrolysis and methanogenesis (Li et al. 2013).

Although all of the treatments gained satisfied methane yield, low daily methane productivity lasted 2 weeks or more for SPV in SD-AD and D-AD, PM in D-AD, CM and SPV+CM in D-AD (Fig. 1b, c). Total ammonia–nitrogen (TAN) content could maintain pH value by contributing the alkalinity to the AD system. However, ammonia inhibition restrained the microbial metabolism, when the concentration reached 4.0 mg/g of TAN or 250 mg/kg of free ammonia (FA) in AD systems (Procházka et al. 2012; Bujoczek et al. 2000). In this study, methane productivity wasn’t restricted when FA value was over 250 mg/kg, but affected when over 380 mg/kg. Thus, a decrease of 32.44–55.34% was found for PM, CM and SPV+CM in D-AD. For SPV mono-digestion in SD-AD and D-AD, the decline of methane yield (based on per unit VS) was most likely attributable to the rapid hydrolysis of carbohydrates in the initial phase of digestion and poor mass transfer capacity in high TS system, which weakened substrate conversion efficiency.

Briefly, the final VFAs/TA values could be a proper parameter to judge the success of AD or not. However, TAN and free ammonia would be more significant indicators to estimate the methane productivity in this study. These results effectively suggested that, in contrast with mono-digestion, co-digestion of SPV and manure balanced the relationship of microbial communities, improved process stability and enhanced the methane production performance under W-AD, SD-AD and D-AD conditions.

### Modified first order model of methane production

Table 4 summarize the results of fitting the modified first order model to digestion data obtained with SPV and animal manure under various TS conditions. The equation describes the conversion constant and methane production potential for the experimental results well with R^2^ values within the range of 0.9715–0.9966. The first order constant (*κ*), as an indicator for substrate biodegradability, reflects the hydrolysis efficiency and metabolic activity. In this study, *κ* value in W-AD ranged from 0.0642 to 0.0961 1/day, in SD-AD ranged from 0.0003 to 0.0953 1/day, and in D-AD ranged from 0.0003 to 0.0796 1/day, which was partial consistency with the earlier work reported by Brown et al. (2012). SPV co-digestion with animal manure could increase the *κ* value, which indicated better degradation efficiency and methane yield. As the increasing of total solid, DM kept the *κ* value more stable than PM and CM, which indicated DM with greater buffer ability to acid than other manure. Interestingly, regardless of substrate types and total solid concentration, obvious reduction of methane yield was found as *κ* value was lower than 0.01 1/day in batch test.

**Table 4:** Parameters of modified first order model from different mixtures of SPV and animal manure in various anaerobic conditions

Samples	W-AD		SD-AD		D-AD	
	*κ*	R^2^	*κ*	R^2^	*κ*	R^2^
SPV	0.0961	0.9799	0.0003	0.9764	0.0006	0.9963
DM	0.0742	0.9936	0.0834	0.9934	0.0751	0.9966
SPV+DM	0.0996	0.9840	0.0953	0.9889	0.0796	0.9915
PM	0.0717	0.9904	0.0394	0.9953	0.0031	0.9859
SPV+PM	0.0848	0.9839	0.0441	0.9908	0.0137	0.9846
CM	0.0642	0.9899	0.0389	0.9924	0.0078	0.9880
SPV+CM	0.0866	0.9819	0.0326	0.9884	0.0003	0.9715

SPV, sweet potato vine; DM, dairy manure; PM, pig manure; CM, chicken manure; W-AD, wet anaerobic digestion; SD-AD, semi-dry anaerobic digestion; D-AD, dry anaerobic digestion

## Discussion

In view of substrate utilization efficiency, the increase of TS concentration had the negative effect to methane production performance. Usually, there are a series of important parameters to evaluate the operation stability, including pH, VFAs, TA and ammonia concentration (Callaghan et al. 2002; Weiland 2010). As shown in Table 3, suitable pH range and VFAs/TA ratio guaranteed gas production efficiency. However, methane productivity was restricted when FA value was over 380 mg/kg in the reactors, which was also supported by Bujoczek et al. (2000).

In this study, co-digestion increased the daily and cumulative methane yield of substrate, and similar result was found by Li et al. (2013). As TS concentration increased, similar trends were found that the decrease of the daily methane yield in SPV+DM digesters was far less than in SPV+PM and SPV+CM systems. Also, weighted EMY was confirmed that co-digestion improved the methane production efficiency, especially in SD-AD and D-AD. Fitting parameters calculated from the modified first order model were further proved co-digestion had better hydrolysis efficiency and metabolic activity.

In conclusion, the maximum methane yield and volumetric production of mono-digestion of SPV were 200.22 mL/g VS_added_ in W-AD and 12.20 L_methane_/L_working_ _volume_ in D-AD. Compared with digestion of SPV as single substrate, co-digestion with manure increased methane yield and volumetric productivity within the range of 14.34–49.11% under different AD conditions. The significant synergistic effects were found in SD-AD and D-AD reactors. FA value of 380 mg/kg was considered as an indicator to decrease process stability, whereas VFAs/TA and pH was in the acceptable range. The mathematical modified first order model was applied to estimate substrate biodegradability and methane production potential well with R^2^ values within the range of 0.9715–0.9966 and conversion constant ranged from 0.0003 to 0.0953 1/day. Co-digestion with SPV and manure improved *κ* value, whereas obvious methane reduction was found as *κ* value was lower than 0.01 1/day. These results offer useful information to future application of anaerobic digestion with SPV and manure as feedstock.

## Abbreviations

AD: anaerobic digestion
W-AD: wet anaerobic digestion
SD-AD: semi-dry anaerobic digestion
D-AD: dry anaerobic digestion
OS: original sludge
CS: centrifuged sludge
SPV: sweet potato vine
CM: chicken manure
DM: dairy manure
PM: pig manure
S/I ratio: substrate/inoculum ratio
TS: total solid
VS: volatile solid
OL: organic loading
TA: total alkalinity
TAN: total ammonia nitrogen
FA: free ammonia
VFAs: volatile fatty acids
EMY: experimental methane yield
